# Genotype-phenotype analysis of von Hippel-Lindau syndrome in Korean families: HIF-α binding site missense mutations elevate age-specific risk for CNS hemangioblastoma

**DOI:** 10.1186/s12881-016-0306-2

**Published:** 2016-07-20

**Authors:** Jee-Soo Lee, Ji-Hyun Lee, Kyu Eun Lee, Jung Hee Kim, Joon Mo Hong, Eun Kyung Ra, Soo Hyun Seo, Seung Jun Lee, Man Jin Kim, Sung Sup Park, Moon-Woo Seong

**Affiliations:** Department of Laboratory Medicine, Seoul National University College of Medicine, 101 Daehak-ro, Jongno-gu, Seoul, 110-744 Korea; Department of Surgery, Seoul National University College of Medicine, 101 Daehak-ro, Jongno-gu, Seoul, 110-744 Korea; Department of Internal Medicine, Seoul National University College of Medicine, 101 Daehak-ro, Jongno-gu, Seoul, 110-744 Korea

**Keywords:** Genotype-phenotype correlation, Hypoxia-inducible factor 1, von Hippel-Lindau disease, von Hippel-Lindau Tumor suppressor protein

## Abstract

**Background:**

von Hippel-Lindau (VHL) disease is a rare hereditary tumor syndrome caused by *VHL* gene mutations that is characterized by heterogeneous phenotypes such as benign/malignant tumors of the central nervous system, retina, kidney, adrenal gland, and pancreas. The genotype-phenotype correlation has not been well characterized in the Korean population so far. Therefore, this study aimed to evaluate the *VHL* mutation spectrum and genotype-phenotype correlations in Korean VHL patients.

**Methods:**

Thirteen unrelated subjects with *VHL* mutations were included. Direct sequencing and multiplex ligation-dependent probe amplification were performed. Consequently, the clinical manifestations and family histories of the subjects were evaluated.

**Results:**

We identified 10 different *VHL* mutations. The c.160_161delAT frameshift mutation was novel. Missense mutations clustered in 2 domains (α domain in exon 1; β domain in exon 3). The most frequently observed mutation was c.208G > A (p.Glu70Lys). Milder phenotypes were observed in subjects with *de novo* mutations. Age-specific risk for CNS hemangioblastoma was significantly higher in subjects carrying missense mutations within the HIF-α binding site (*P* < 0.05).

**Conclusions:**

This study provides insight into the genotype-phenotype correlation in that amino acid substitutions in the HIF-α binding site may predispose patients to age-related risks of CNS hemangioblastoma.

## Background

von Hippel-Lindau disease (VHL) (OMIM no.193300) is an uncommon autosomal dominant cancer syndrome resulting from mutations in the *VHL* tumor suppressor gene. The reported incidence of VHL is about 1 in 36,000–53,000 births worldwide, with age-dependent high penetrance [1–3]. The VHL-related tumors include central nervous system (CNS) hemangioblastomas (CHBs), retinal hemangioblastomas (RHBs), pheochromocytomas (PCCs), renal cell carcinomas (RCCs), endolymphatic sac tumors (ESTs), epididymal cystadenomas, and broad ligament cystadenomas. Additionally, VHL patients often exhibit multiple cysts in various organs including the pancreas and kidney [4].

*VHL* is located on chromosome 3p25.3, and it was first identified in 1993 [5]. The encoded VHL protein (pVHL) forms a complex with elongation factor C and B (elongin C/B), which, along with cullin2 (CUL2) and RING finger protein (RBX1), constitutes the VCB-CR complex. When stabilized, the VCB-CR complex successfully regulates hypoxia-inducible factor α (HIF-α); the prolyl hydroxylated HIF-α directly binds to the β domain of pVHL, and is consequently targeted for polyubiquitination and proteasomal degradation [2]. When pVHL does not regulate HIF-α, the stabilized HIF-α accumulates and stimulates pro-angiogenic factors, such as vascular endothelial growth factor (VEGF) and platelet-derived growth factor β (PDGFB) accelerating tumorigenesis [6].

VHL is clinically classified into 4 phenotypic categories: Type 1 does not include PCC; Type 2A includes PCC but not RCC; Type 2B includes both PCC and RCC; and Type 2C is associated with PCC as the sole manifestation.

Genotype-phenotype correlation studies have provided critical strategies for prophylactic surveillance and genetic counseling of presymptomatic members in VHL families [7, 8]. While the *VHL* genotype-phenotype correlation has been investigated in Western countries, the correlations in Korean populations have not yet been well studied [4, 9, 10].

In this study, we investigated the *VHL* mutation spectrum in Korean patients and evaluated their genotype-specific phenotypes.

## Methods

### Patients

**Fig. 1:**
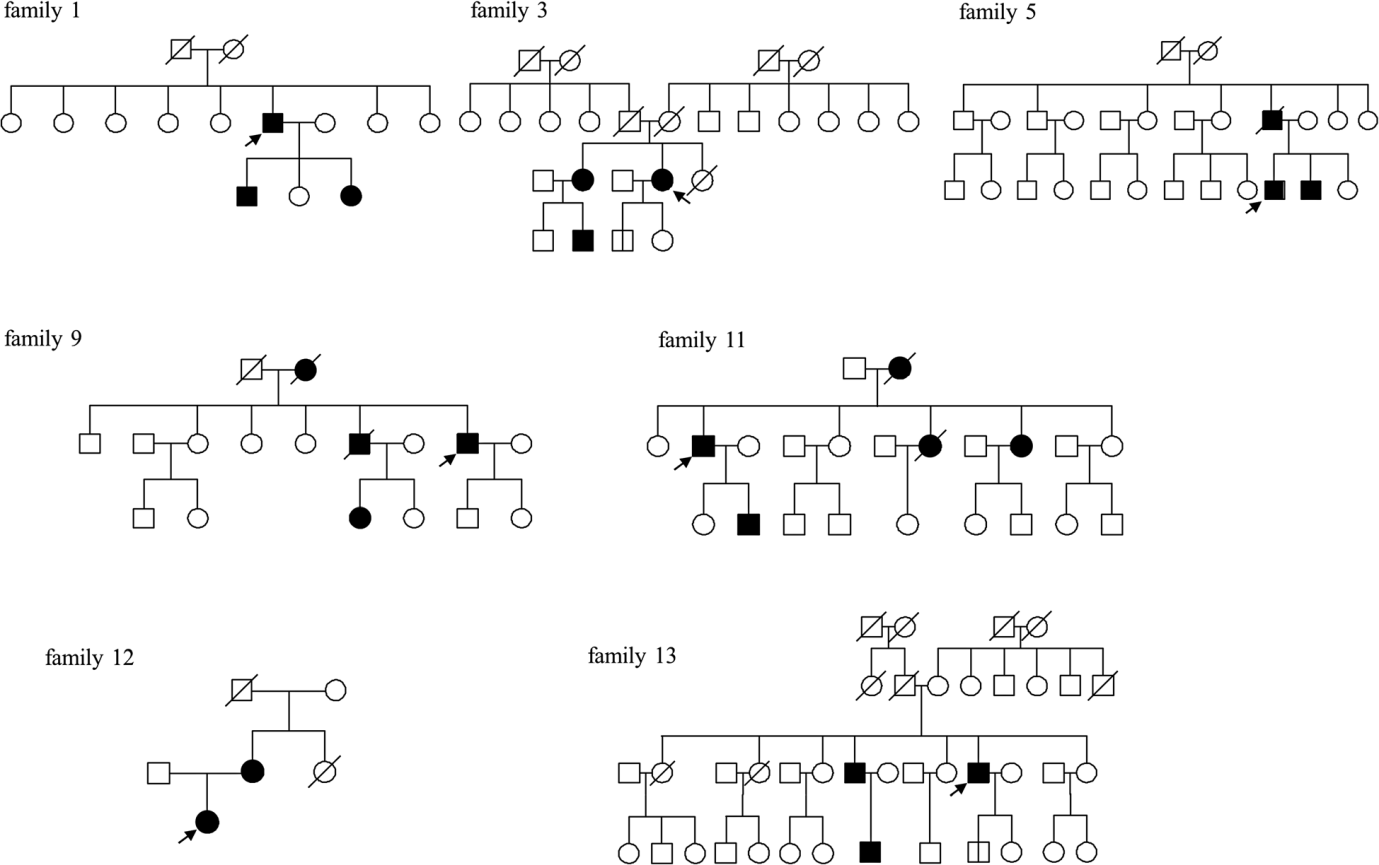
Pedigrees of VHL families (family 1, 3, 5, 9, 11, 12 and 13). *Black* symbols represent the affected subjects. Probands are marked with *arrows*. A further family history assessment of family 4 was not available

Thirteen unrelated patients with germline *VHL* mutations who were diagnosed with VHL disease were evaluated. Patient medical records such as MRI of the brain and the whole spine with contrast, eye examinations, and imaging scans of the abdomen were retrospectively reviewed. In addition, patients were interviewed to obtain information regarding their family history, through which three-generation pedigree data was collected (Fig. 1). The overall observation period ranged from 1988 to 2015. The average follow-up of the subjects was 11.0 years (range: 2–28 years). The study was approved by the institutional review board of the Seoul National University Hospital. Informed consent was obtained from all participants or their parents.

### Genetic analysis

Genomic DNA was extracted from peripheral blood using the Gentra Puregene Blood kit (Gentra Systems, Minneapolis, MN, USA), according to the manufacturer’s instructions. All coding exons and flanking intronic regions were amplified by PCR using primers specific for VHL exons 1–3. The amplified product was directly sequenced with an ABI PRISM 3730xl DNA Analyzer (Applied Biosystems, Foster City, CA, USA) using the BigDye Terminator v.3.1 Cycle Sequencing Kit (Applied Biosystems).

For screening exon deletions and duplications, multiplex ligation-dependent probe amplification (MLPA) P016-C1/P16-C2 kits (MRC-Holland, Amsterdam, Netherlands) were used. DNA denaturation, probe-target sequence hybridization, probe ligation, and PCR amplification of ligated probes were performed according to the manufacturer’s instructions. The products were loaded onto ABI PRISM 3130xl DNA Analyzer (Applied Biosystems) and analyzed by GeneMarker software version 1.51 (SoftGenetics, LLC, State College, PA, USA).

### Statistics

The tumors in the 2 different *VHL* mutation groups (inherited mutations vs. *de novo* mutations) were compared using the Mann–Whitney *U* test. Kaplan–Meier estimates with log-rank test were used to calculate the age-related penetrance of VHL-associated tumors. *P* values less than 0.05 were considered statistically significant. Data was analyzed using SPSS version 21.0 software (IBM-SPSS Inc, Chicago, IL, USA).

## Results

### Cases

Patient 1, a 49-year old male (Table 1), presented with gait disturbance and vertigo. MRI evaluation revealed hemangioblastoma at the right superior cerebellum. A year after excising the mass, bilateral 2.2–4.0 cm tumors were found in both kidneys. Eye examination revealed a hemangioma-like lesion in the right eye. In addition, CT of the abdomen revealed a pancreatic neuroendocrine tumor (NET). Genetic testing showed that the patient harbored a novel frameshift mutation, p.Met54Glyfs*77, in the *VHL* gene. One of his daughters and his son were heterozygous for the same mutation. His 35 year-old daughter had 3 types of VHL-related tumors: Cerebellar hemangioblastoma, RCC in the right kidney lower pole, and pancreatic NET which invaded the superior mesenteric artery and superior mesenteric vein. His son, who was 38 years old, visited an emergency room presenting with a generalized tonic-clonic seizure. On evaluation, the brain MRI showed a well-defined mass in the left parietal area, which was pathologically found to be a meningioma.

**Table 1:** Germline *VHL* mutation and its phenotypes in 13 VHL families

Family	Sex	FHx	Exon	Nucleotide change	AA change	VHL type	CHB	RHB	RCC	PCC	PC	KC	Others	Reference
1	M	Proband	1	c.160_161delAT	p.Met54Glyfs*77	1	49	64	51, B	–	64, M	–		–
	M	Son	1	c.160_161delAT	p.Met54Glyfs*77	1	–	–	–	–	38, M	38, B	+	–
	F	Daughter	1	c.160_161delAT	p.Met54Glyfs*77	1	35	–	35	–	35, M	35, B		–
2	M	–	1	c.208G > A	p.Glu70Lys	1	25	–	–	–	–	–		14, 19
3	F	Proband	1	c.208G > A	p.Glu70Lys	1	–	46, B	–	–	–	–		14, 19
	M	Son	1	c.208G > A	p.Glu70Lys	–	–	–	–	–	–	–		14, 19
4	F	Proband	1	c.208G > A	p.Glu70Lys	1	–	20, B	–	–	–	–		14, 19
	F	Mother	NA	NA	NA	1	–	20, B	–	–	–	–		
5	M	Proband	1	c.227_229delTCT	p.Phe76del	1	12	–	19, B	–	13, M	–		4
	M	Brother	NA	NA	NA	1	19	–	18, B	–	23, M	19, B		
	F	Sister	IVS1	c.340 + 5G > C		–	–	–	–	–	–	–	+	
		Father	NA	NA	NA	2B	27	–	44,B	44	44,M	44,B		
6	M	–	1	c.232A > G	p.Asn78Asp	1	11	–	–	–	20, M	20		15
7	M	–	1	c.233A > G	p.Asn78Ser	1	14	14	–	–	14, M	14, B		4
8	M	–	IVS2	c.464-1G > T		1	38	–	–	–	38, M	38		4
9	M	Proband	3	c.499C > T	p.Arg167Trp	2B	47	–	47	47	47, M	48		4
	F	Niece	3	c.499C > T	p.Arg167Trp	1	14	26, B	32	–	26, M	32, B		4
10	F	–	3	c.500G > A	p.Arg167Gln	1	59	–	–	–	59, M	59, B		4
11	M	Proband	3	c.500G > A	p.Arg167Gln	1	36	41, B	48, B	–	–	48, B		4
	M	Son	3	c.500G > A	p.Arg167Gln	1	15	–	–	–	–	–		4
12	F	Proband	3	c.592delC	p.Leu198Trpfs*4	2C	–	–	–	24	21, M	–		19
	F	Mother	3	c.592delC	p.Leu198Trpfs*4	2C	–	–	–	27	48, M	–	+	19
13	M	Proband		Exon2, 3 deletion		1	32	44	44, B	–	41	–		4
	M	Son		Exon2, 3 deletion		–	–	–	–	–	–	–		4
	M	Brother		Exon2, 3 deletion		1	29	52	43, B	–	43, M	43, B		4
	M	Nephew	NA	NA	NA	1	22	18	29, B	–	18, M	–		

*Abbreviations: CHB, CNS* hemangioblastoma, *RHB* retinal hemangioblastoma, *RCC* renal cell carcinoma, *PCC* pheochromocytoma, *PC* pancreas lesion (pancreatic cyst or pancreatic tumor), *KC* renal cyst, Other includes developmental venous anomaly or meningioma, *B* bilateral, *M* multiple, *AA* amino acid, *FHx* family history, *NA* not tested

Patient 2 was a 25-year-old male (Table 1) who reported frequent headaches and dizziness. MRI of the brain revealed a 0.9-cm nodular lesion at the medullocervical junctional level. This patient underwent midline suboccipital craniotomy. A heterozygous missense mutation was identified in the *VHL* gene: p.Glu70Lys. There was no family history. This patient regularly visited the clinic, and no other VHL-related tumor developed.

Patient 3 was a 46-year-old female who was the index patient in family 3 (Table 1). She visited the clinic with a 3-month history of blurred eye vision in her left eye. Examination revealed RHB in her left eye. *VHL* analysis revealed the heterozygous missense mutation p.Glu70Lys. Since her sister had suffered from RHB, the mutation was considered to be passed down from her parents. Her son, a 19-year-old male, inherited the same mutation, but no VHL-related symptoms were present. He underwent surveillance for VHL manifestations. RHB was the only VHL-related phenotype in this family.

Patient 4 was a 20-year-old female (Fig. 1). Through routine eye examination, multiple RHBs in both eyes were found. We analyzed the *VHL* gene and found that the patient was heterozygous for the p.Glu70Lys mutation. Her mother had been treated for RHB but refused to undergo genetic analysis. Further genetic testing and family history assessment was unavailable for this family. No other VHL-associated symptoms were observed in patient 4 and her mother.

In family 5, two siblings (Table 1) underwent surgical resection for CHB at the ages of 12 and 19. Abdominal CT revealed RCCs in these siblings. The family history revealed VHL manifestations in their father who died in 2013 and had RCC, PCC, and CHB (Table 1). The proband (patient 5) harbored an in-frame deletion p.Phe76del affecting exon 1 in the *VHL* gene. This in-frame deletion appeared to run in this family.

Patient 6 described nausea and vomiting at the age of 11. Imaging studies showed multiple enhancing soft tissue masses on the cerebellar tonsil, cerebellar hemisphere, and cervical spinal cord. CT of the abdomen revealed pancreatic and renal cysts along with pancreatic NET. *VHL* analysis revealed a heterozygous substitution c.232A > G, which leads to the p.Asn78Asp mutation. This patient had no family history.

Patient 7 was 14-year-old male who experienced frequent abdominal distension and intermittent headaches. At a routine health check-up, multiple pancreatic cystic lesions and RHB in the temporal disc side of his left eye were found. MRI of the brain showed a tiny well-enhanced nodule at the cervicomedullary junction with an adjacent prominent vascular structure. The features of this patient’s presentation were indicative of VHL. Although this patient had no family history, a heterozygous missense *VHL* mutation, p.Asn78Ser, was identified in patient 7.

Patient 8, a 38-year-old male, showed mild imbalance with gait difficulty. MRI of the brain revealed a 3.5-cm cystic mass in the cerebellum, which was pathologically found to be hemangioblastoma. When this patient underwent CT, which included the pancreas and kidney, multiple pancreatic cysts and renal cysts were found. A *VHL* c.464-1G > T splicing mutation was detected in patient 8 and there was no family history of VHL disease in his family.

Patient 9 was a 47-year-old male (Table 1). He presented with dizziness and headache, which he had experienced for the 3 months prior to examination. After systemic examination, multiple VHL related tumors were revealed, including RCC, PCC, and CHB. *VHL* analysis detected a heterozygous missense mutation, p.Arg167Trp. He had a family history of VHL-related tumors, including CHB and RCC. His brother was diagnosed with CHB and his niece was diagnosed with CHB, RHB and RCC. The same mutation was identified in his niece.

Patient 10 (Table 1), a 59-year-old female, presented with indigestion, anorexia, and nausea. This patient underwent abdominal CT; multiple renal cysts, a hypervascular mass in the pancreatic body, and a cystic mass in the pancreatic head and tail were found. Pylorus preserving pancreaticoduodenectomy was performed in this patient. Two months after surgery, gait disturbance appeared. MRI of the brain revealed multiple enhancing masses in both cerebellums, which were pathologically found to be hemangioblastoma. Based on suspicion of VHL, we sequenced the *VHL* gene in this patient; a heterozygous missense *VHL* mutation, p.Arg167Gln, was confirmed. None of her family members were affected.

Patient 11 (Table 1) was diagnosed with VHL at the age of 36 years, while his son was diagnosed at the age of 15 years. Patient 11 presented with nausea, vomiting, and left-side weakness, developed within 2 days. MR brain images revealed a hemangioblastoma-like cystic mass in the cerebellum. He therefore underwent cerebellar tumor resection. When he was under observation, RHB and RCC were detected at the ages of 41 and 48 years, respectively. Patient 11 and his son shared a heterozygous missense mutation, p.Arg167Gln, in the *VHL* gene. The mother and sisters of patient 11 were also suspected to have *VHL* mutations, but their DNA was not evaluated.

Patient 12 and her mother were diagnosed with pheochromocytoma at the ages of 24 and 27 years, respectively (Table 1). A frameshift mutation, p. Leu198Trpfs*4, in the *VHL* gene was detected in these 2 subjects.

Patient 13, his brother, and his nephew were diagnosed with VHL. RCC and CHB were passed from generation to generation (Table 1). A large deletion spanning exons 2–3 in the *VHL* gene was detected in patient 13, his brother, and his asymptomatic son.

### Clinical characteristics of patients

A total of 13 unrelated probands (9 males and 4 females) were analyzed in our study (Table 1). Their mean age at first manifestation was 31.5 ± 15.7 years. CHB was the most common presenting phenotype (*n* = 10, mean age at diagnosis 32.3 ± 16.7 years), followed by pancreatic lesions (*n* = 9, mean age at diagnosis 35.2 ± 19.2 years), renal cysts (*n* = 6, mean age at diagnosis 37.8 ± 17.6 years), RHB (*n* = 6, mean age at diagnosis 38.2 ± 18.4 years), RCC (*n* = 5, mean age at diagnosis 41.8 ± 13.0 years), and PCC (*n* = 2, mean age at diagnosis 35.5 ± 16.3 years). Excluding PCC, bilateral or multiple lesions frequently occurred in VHL related tumors: 80.0 % in CHB, 50.0 % in RHB, 80.0 % in RCC, 88.9 % in pancreatic lesions, and 50.0 % in renal cysts. Among the 13 patients who were carriers of *VHL* mutations, 8 (61.5 %) had a family history of the disease while the other 5 (38.5 %) had no VHL family history; thus, the latter group was considered as having *de novo* germline *VHL* mutations.

### Mutation distribution in VHL disease

In our study, 10 different VHL germline mutations were confirmed in 13 unrelated VHL patients (Table 1). Our data showed that 61.5 % (8/13) of probands had missense mutations and 15.4 % (2/13) had frameshift mutations. In-frame deletion, splicing mutation, and exon deletion were found in each 7.7 % (1/13) of the probands. The novel mutation c.160_161delAT (p. Met54Glyfs*77) was identified.

**Fig. 2:**
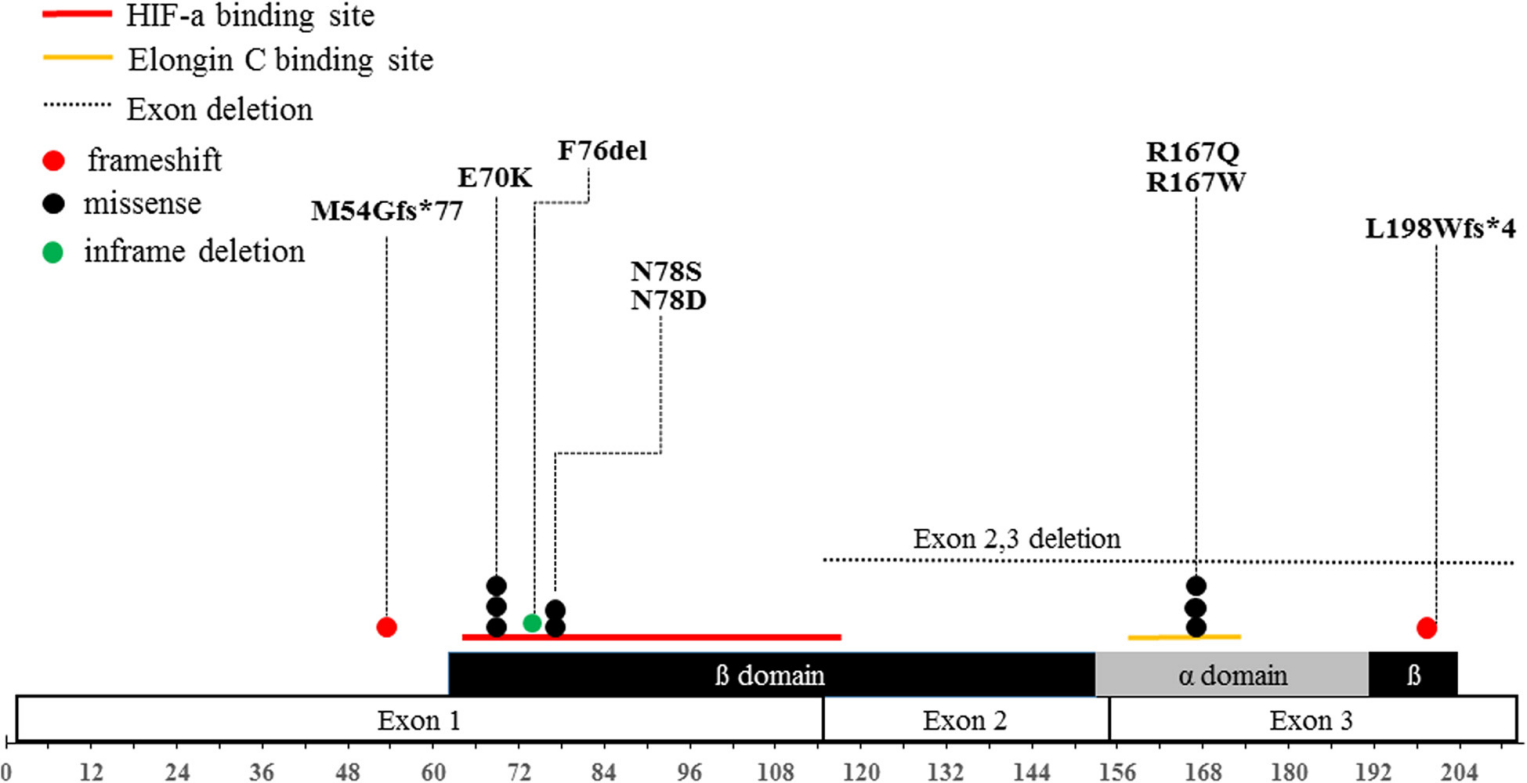
Distribution of germline mutations in VHL patients. Three exons are shown by boxes and α/β domains are indicated by colored boxes. The horizontal bars indicate binding domains. A splice site mutation, c.464-1G > T is not shown in this figure

Mutations were mainly concentrated in 2 domains, α and β, while no mutation was found in the codons from 1 to 53 (Fig. 2).

Missense mutations (p.Arg167Trp and p.Arg167Gln) in codon 167, in which VHL mutations frequently occur throughout all ethnic groups, were also observed in Korean population: p.Arg167Trp, in 1 proband (7.7 %) and p.Arg167Gln, in 2 unrelated probands (15.4 %). Interestingly, the most frequently observed mutation was c.208G > A (p.Glu70Lys), which occurred in 3 unrelated probands (23.1 %).

### Genotype-phenotype correlations

Three subjects carrying the c.208G > A (p.Glu70Lys) mutation manifested only CHB or RHB; 1 patient had only CHB and the other 2 patients manifested only RHB. No other phenotype was observed in these 3 patients. Notably, RHB developed more frequently in patients with c.208G > A mutation (2/3, 66.7 %) than in patients with other mutations (4/10, 40.0 %).

**Fig. 3:**
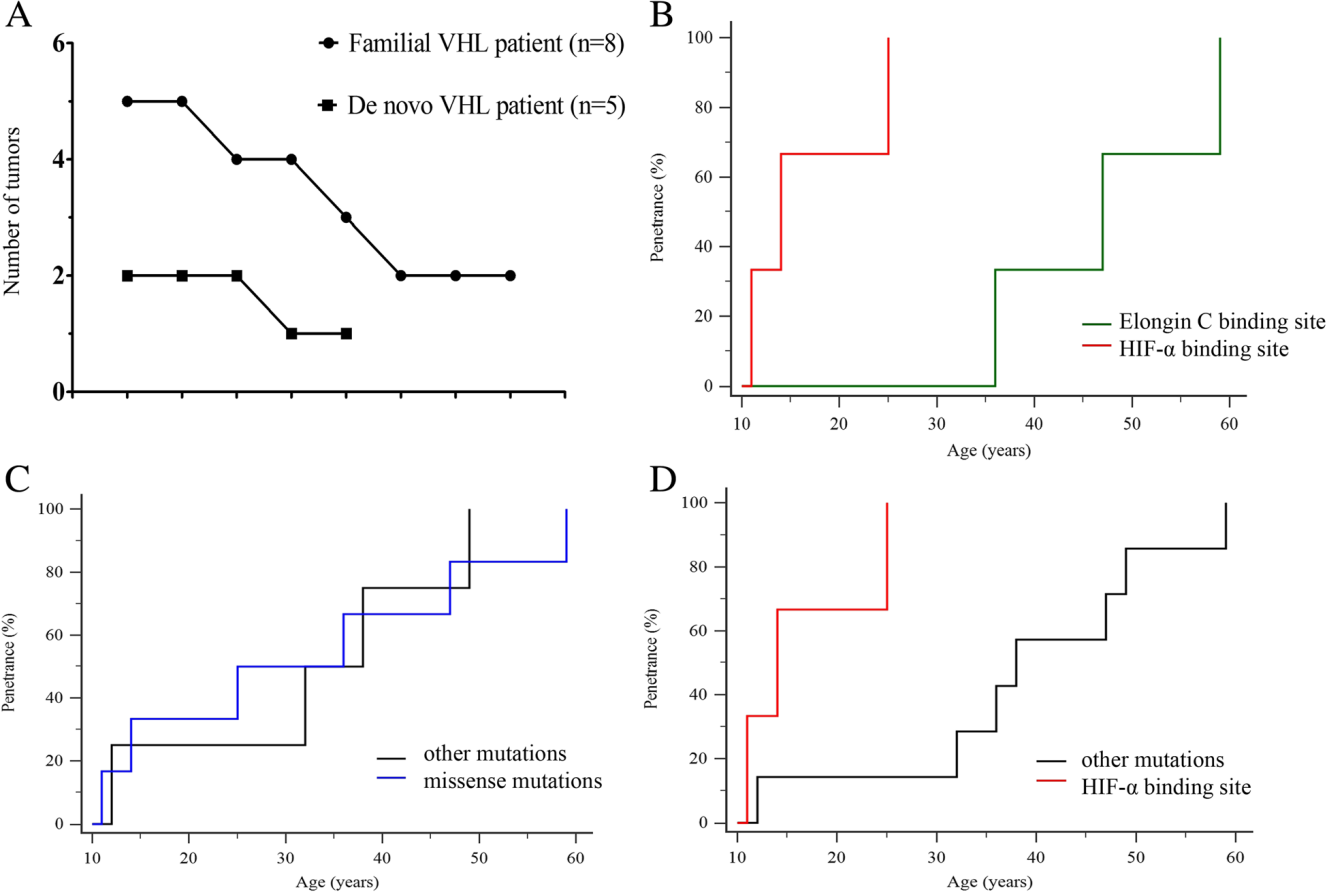
Phenotypic traits of Korean VHL patients. **a** Number of VHL-related tumors in 2 groups of VHL patients (*de novo* mutation vs. familial mutation). The patients are represented on the x-axis. **b**–**d** Age-related penetrance of CHB in VHL mutations. **b** HIF-α binding site vs. elongin C binding site. **c** Missense mutations vs. other mutations. **d** Missense mutations within the HIF-α binding site vs. other mutations

Patients with *de novo VHL* germline mutation had significantly lower number of tumors (mean 1.6 tumors) than patients with a family history of the mutation (mean 3.4 tumors) (*P* = 0.007, Fig. 3a). RCC was not present in patients with *de novo* VHL mutations (Table 1).

All of the pathogenic missense mutations were concentrated within either the HIF-α binding site (residues 65–117) or the elongin C binding site (residues 158–170) (Fig. 2). With regard to the age-related penetrance, patients carrying amino acid substitutions in HIF-α binding site had a significantly higher risk for CHB than patients carrying missense mutations in another site (*P* = 0.025) (Fig. 3b). Interestingly, while the age-related risk between the group of patients with an overall missense mutation and other patients was not significantly different (Fig. 3c), missense mutations especially occurring at the HIF-α binding site increased the age-specific penetrance for CHB (Fig. 3d).

Our mutation profile did not represent the correlation between type 2 VHL and missense mutations: PCC was found in 2 subjects, who were carrying a missense mutation c.499C > T (p.Arg167Trp) and a frameshift mutation c.592delC (p.Leu198Trpfs*4). The c.592delC mutation was predicted to interrupt the 198^th^ codon in *VHL* coding regions (codons 1–213).

We did not find any association between RCC and truncating VHL mutations. The incidence of RCC was not significantly higher in patients with truncating VHL mutations than in those missense mutations (66.7 % vs. 33.3 %, *P* = 0.523). Furthermore, age-related RCC risk between patients with truncating mutations and those with missense mutations was not significantly different (*P* = 0.455).

## Discussion

This study describes the unique phenotypic traits and mutation spectrum of VHL syndrome in 13 Korean families. Our data showing that missense mutations were all clustered in either HIF-α or elongin C binding sites supports previous findings that these two binding sites have a role in VHL protein function [2, 4, 11]. Previous studies suggested that dysregulated HIF-α is the chief causative factor of hemangioblastoma, a highly vascular tumor [2, 4]. We speculate that missense mutations disrupting HIF-α binding can increase the risk for early-age onset of CHB. It has previously been reported that the mean age at diagnosis for VHL-associated CHB is 32 years [12], while our data showed that CHB patients harboring missense mutations in the HIF-α binding site developed CHB at an early age (mean 16.7 years). The HIF-α binding site mutations in our data include Asn78Ser, Asn78Asp, and Glu70Lys. All of the HIF-α binding site missense mutations observed in this study were previously identified to reduce HIF-α degradation [13–15]. Asn78Ser disables binding as well as ubiquitination of HIF-α, while it retains binding of elongin C, both in vitro and in vivo [13]. The pVHL L1 loop mutant Glu70Lys disrupts HIF-α binding activity and HIF-2a degradation in vivo [14]. However, Arg167Gln is located within the elongin C binding site, which only eliminates the elongin C binding. The Arg167Gln mutant still has remnant VHL–elongin BC complex and retains wild-type levels of HIF1-α regulation [16]. Since HIF-α dysregulation is a critical step in the development of hemangiomas, disruption of functional pVHL that regulates HIF-α can accelerate CHB progression. Inhibition of HIF-α degradation may lead to overexpression of hypoxia-inducible mRNAs such as VEGF, which promotes tumor angiogenesis, especially in highly vascularized tumors, including CHB [2]. Thus, patients harboring HIF-α binding site missense mutations may show accelerated onset of CHB, manifested by rapid rate of tumor growth.

Interestingly, two patients with elongin C binding site mutations also presented CHB onset at an early age (at 14 and at 15 years, respectively) (Table 1). These two patients were the niece and the son of the study subjects, probands of family 9 and 11, respectively. The onset age may be earlier in offspring of the probands than in the probands themselves. A previous study suggested that this is expected in families with VHL disease [17], supporting our findings.

Our findings suggest that categorizing missense mutations according to their effect on HIF-α regulation enables strict follow-up of high-risk patients for early-onset hemangioblastomas.

Our results show that 38.5 % (5/13) of Korean VHL patients were classified as *de novo* patients. This *de novo* mutation is probably due to sporadic germline *VHL* mutations or can be inherited from a mosaic parent [4]. Sgambati et al. [8] reported 4.8 % rate of mosaicism in asymptomatic parents of *de novo* VHL patients, a finding that is similar to that reported in families affected with Type 2 neurofibromatosis (NF2). Decker et al. [18] reported that *de novo* mutations may have up to 20 % incidence. Recently, a higher prevalence of *de novo VHL* germline mutations (56.3 %) was reported in China [19]. Notably, we observed a significantly lower number of VHL-related tumors in sporadic VHL patients than in patients with a family history of VHL (Fig. 3a). This finding reveals that the *de novo* VHL patients have mild phenotypes; the proband first affected in the family is most likely to carry mosaic VHL mutations.

The high incidence of Glu70Lys mutation observed in this study (frequency of 23.1 %) and its limited range of manifestations (either CHB or RHB) corresponds well with the findings of Hwang et al. [20]. While previous VHL studies described codons 167, 76, 98, 65, and 78 as frequently mutated regions, there are only a few reports of Glu70Lys mutation in different ethnic groups [21, 22]. Mettu et al. [21] reported that among the 412 VHL patients studied, only one patient (frequency of 0.24 %) carried the Glu70Lys mutation. Since this mutation is highly frequent in Korean populations [20], codon 70 seems to be the hot spot region in the ethnic specific mutational spectrum.

It has been reported that VHL type 2 is exclusively correlated with missense mutations that cause changes in the amino acids surface of the pVHL, and these proteins retain its ability to downregulate HIF-α [4, 6, 18]. Interestingly, the results of the present study demonstrate that PCC does not always occur in association with a single amino acid change. The p.Leu198Trpfs*4 mutation was observed in 1 PCC patient. Since this frameshift mutation is located in the 3′-most exon, it is unable to cause a complete loss of pVHL function. These findings are consistent with the hypothesis that some retention of pVHL function is critical for tumorigenesis in PCC [23].

Notably, we identified a novel frameshift mutation in Korean VHL patients, c.160_161delAT (p.Met54Glyfs*77), which occurs in codon 54 and induces a premature stop at codon 130.

The major limitations of this study were the small number of patients analyzed and the examination of only one ethnic group, limiting the generalization of the study results. However, our findings revealed that a missense mutation in the HIF-α binding site serves as a distinct predictor for early onset CHB. This study enhances the efficacy of the surveillance of VHL patients.

## Conclusions

The present study evaluated the genotype-specific phenotypes of VHL in the Korean population. The findings in our study will provide insights for the genetic counseling and management of families suffering from VHL. Missense mutations that disrupt HIF-α binding may predispose to early-onset CHB in Korean VHL patients. It is recommended that patients carrying these mutations undergo aggressive screening and early treatment for CHB to achieve a better treatment outcome.

## Abbreviations

CHB, hemangioblastoma; CNS, central nervous system; CUL2, cullin2; EST, endolymphatic sac tumors; HIF- α, hypoxia-inducible factor α; KC, renal cyst; MLPA, multiplex ligation-dependent probe amplification; PC, pancreas lesion; PCC, pheochromocytomas; PDGFB, platelet-derived growth factor β; pVHL, VHL protein; RBX1, RING finger protein; RCC, renal cell carcinomas; RHB, retinal hemangioblastomas; VEGF, vascular endothelial growth factor; VHL, von Hippel-Lindau disease
